# Successful breeding predicts divorce in plovers

**DOI:** 10.1038/s41598-020-72521-6

**Published:** 2020-09-23

**Authors:** Naerhulan Halimubieke, Krisztina Kupán, José O. Valdebenito, Vojtěch Kubelka, María Cristina Carmona-Isunza, Daniel Burgas, Daniel Catlin, James J. H. St Clair, Jonathan Cohen, Jordi Figuerola, Maï Yasué, Matthew Johnson, Mauro Mencarelli, Medardo Cruz-López, Michelle Stantial, Michael A. Weston, Penn Lloyd, Pinjia Que, Tomás Montalvo, Udita Bansal, Grant C. McDonald, Yang Liu, András Kosztolányi, Tamás Székely

**Affiliations:** 1 Milner Centre for Evolution, Department of Biology and Biochemistry, University of Bath, Bath, UK; 2 Behaviour Genetics and Evolutionary Ecology Research Group, Max Planck Institute for Ornithology, Seewiesen, Germany; 3 Department of Evolutionary Zoology and Human Biology, University of Debrecen, Debrecen, Hungary; 4 Department of Animal and Plant Sciences, University of Sheffield, Alfred Denny Building, Western Bank, Sheffield, UK; 5 Department of Biodiversity Research, Global Change Research Institute, Czech Academy of Sciences, Brno, Czech Republic; 6 Departamento de Ecología Evolutiva, Instituto de Ecología, Universidad Nacional Autónoma de México, Ciudad de México, México; 7 Department of Biological and Environmental Science, University of Jyväskylä, Jyväskylä, Finland; 8 Department of Fish and Wildlife Conservation, Virginia Tech, Blackburg, USA; 9 Centre for Biological Diversity, School of Biology, University of St Andrews, St Andrews, UK; 10 Department of Environmental and Forest Biology, SUNY College of Environmental Science and Forestry, Syracuse, USA; 11 Department of Wetland Ecology, Estación Biológica de Doñana, Sevilla, Spain; 12 Quest University Canada, Squamish, Canada; 13 Forest Supervisor’s Office, USDA Forest Service, Plumas National Forest, Quincy, CA USA; 14 Associazione ARCA, Senigallia-Anoca, Italy; 15 Posgrado en Ciencias del Mar Y Limnología, Universidad Nacional Autónoma de México, Ciudad Universitaria, Cd. México, Mexico; 16 School of Life and Environmental Sciences, Faculty of Science, Engineering and the Built Environment, Deakin University, Burwood, Australia; 17 FitzPatrick Institute, DST/NRF Centre of Excellence, University of Cape Town, Cape Town, South Africa; 18 Ministry of Education Key Laboratory for Biodiversity Science and Ecological Engineering, College of Life Sciences, Beijing Normal University, Beijing, China; 19 Chengdu Research Base of Giant Panda Breeding, Chengdu, China; 20 Sichuan Key Laboratory of Conservation Biology for Endangered Wildlife, Chengdu, China; 21 Sichuan Academy of Giant Panda, Chengdu, China; 22 Servei de Vigilancia I Control de Plagues Urbanes, Agencia de Salud Pública de Barcelona, Barcelona, Spain; 23 Centre for Ecological Sciences, Indian Institute of Science, Bengaluru, India; 24 Department of Ecology, University of Veterinary Medicine Budapest, Budapest, Hungary; 25 Department of Zoology, Edward Grey Institute, University of Oxford, Oxford, UK; 26 State Key Laboratory of Biocontrol, School of Ecology/School of Life Sciences, Sun Yat-Sen University, Shenzhen, China

**Keywords:** Ecology, Evolution, Zoology

## Abstract

When individuals breed more than once, parents are faced with the choice of whether to re-mate with their old partner or divorce and select a new mate. Evolutionary theory predicts that, following successful reproduction with a given partner, that partner should be retained for future reproduction. However, recent work in a polygamous bird, has instead indicated that successful parents divorced more often than failed breeders (Halimubieke et al. in Ecol Evol 9:10734–10745, 2019), because one parent can benefit by mating with a new partner and reproducing shortly after divorce. Here we investigate whether successful breeding predicts divorce using data from 14 well-monitored populations of plovers (*Charadrius* spp.). We show that successful nesting leads to divorce, whereas nest failure leads to retention of the mate for follow-up breeding. Plovers that divorced their partners and simultaneously deserted their broods produced more offspring within a season than parents that retained their mate. Our work provides a counterpoint to theoretical expectations that divorce is triggered by low reproductive success, and supports adaptive explanations of divorce as a strategy to improve individual reproductive success. In addition, we show that temperature may modulate these costs and benefits, and contribute to dynamic variation in patterns of divorce across plover breeding systems.

## Introduction

The decision to retain or divorce a mate between successive breeding events is an important aspect of mating systems^2^, with direct implications for reproductive success and subsequent survival of the parents^3–7^. Mate fidelity, defined as retaining the same mate for subsequent breeding attempt(s), is commonly observed in a variety of taxa^8,9^. Mate fidelity varies widely among species in terms of duration, with some exhibiting short-term mate fidelity within a single season, in which an individual remains faithful to a mate throughout one breeding season and initiates another breeding season with a new mate while the old partner is still alive, whereas other species show long-term (i.e. between seasons) or even life-time mate fidelity^8–13^. Understanding the drivers of interspecific variation in mate fidelity is thus crucial to understand the evolutionary diversity of animal mating systems.

Various factors have been proposed to explain variation in mate fidelity across taxa. The abiotic environment (such as temperature and precipitation) often shapes mating decisions. Variation the in abiotic environment may affect resource availability and the duration of suitable breeding periods, creating different ecological constraints that may limit or promote mate fidelity^14–16^. For example, arctic bird species have typically short breeding seasons due to the harsh and stochastic environmental conditions, and tend to exhibit high fidelity to a mate, which is likely to improve offspring survival^17,18^. In contrast, mild environments in temperate and tropical regions tend to provide a more prolonged breeding season so that an individual might initiate multiple clutches with the same or different mates^1,19,20^. The influence of environmental conditions on mating decisions has been observed in a variety of taxa including flies, fish, frogs and birds^21–23^. Aspects of the social environment, such as adult sex ratio (ASR) are also known to influence mating decisions^24^. In species or populations with a biased ASR, the rare sex is more likely to initiate divorce since the rare sex has higher mate availability than the common sex (e.g. frogs^24^, birds^25,26^). Life history traits may also influence rates of mate fidelity; for instance, species with a high divorce rate have a high mortality rate, whereas species with high adult survival rate (or long-lived species) tend to retain the same mates from year to year^27,28^, suggesting that survival rate or longevity predict mating decisions. Although life history theory predicts that large body size is usually related to high survival rate and longevity^10^, there is sparse evidence to show whether larger species exhibit stronger mate fidelity than smaller ones^10,28^. Sexual size dimorphism (SSD) may also relate to patterns of mate fidelity, since more exaggerated SSD may reflect more intense sexual selection and polygamy^29^. Other life history traits (e.g. age of first reproduction, life span) have also been proposed to be linked to mate fidelity^30,31^.

Several hypotheses have been put forward to explain variation in mate fidelity, emphasizing the costs and benefits of individual mating decisions, and their relationship with breeding success or breeding time. The “*fast-track hypothesis*” suggests that individuals retain a mate to reduce the time and energy costs of searching for a new mate^32,33^. The “*mate familiarity hypothesis*” highlights that retaining a mate could improve breeding performance by enhancing coordination between parents and thereby improving reproductive success^13,34,35^. In contrast, changing a mate may be beneficial in some long-lived species, as individuals may divorce their current partner to mate with relatively higher quality or more compatible partners in order to improve breeding success (“*incompatibility hypothesis*”^36^; also see^37^); as a corollary, successful breeding pairs are more likely to stay together for future breeding attempts^11,38,39^. It has also been suggested that divorcing and rapidly changing a mate may be favoured by some species in order to make the most out of a restricted time budget (e.g. short life span or short breeding season)^1,40^.

Mating decisions are also associated with breeding dispersal, i.e. the movement of an adult from one breeding location to another between consecutive breeding attempts within a breeding year^41,42^. Breeding dispersal may exhibit sex differences as males and females can adopt different mating strategies, for instance, the more polygamous sex is more likely to disperse farther to find new mating partners than the less polygamous sex^41,43–45^. Studies also suggest that mate fidelity can be a by-product of site fidelity^28,46^, and conversely, mate change can be a result of changing nest sites^47,48^. Nonetheless, studies of mate fidelity mostly centre around socially monogamous bird species between years^28,49^, and studies that investigate mate fidelity in multiple species and populations that exhibit variable duration of pair-bonds within a single breeding season are scarce^1^.

Here we focus on *Charadrius* plovers—small ground-nesting shorebirds—for four reasons. First, they exhibit intra- and interspecific variation in several behavioural, ecological, demographic and life history traits^16,45^, making them an excellent model system for addressing mate choice decisions. Second, plovers are globally distributed, breeding on all continents except Antarctica, providing an excellent opportunity to conduct a geographically large-scale study^16,45^. Third, they display flexible mating systems including short-term within-year pair-bonds. In some plover populations, both males and females may have up to four breeding attempts with the same or different mate sequentially within a single season^45,50,51^. Finally, their breeding biology is well characterised: plovers typically lay two to four eggs (depending on the species) in poorly insulated nest scrapes with both parents typically providing care during the incubation stage^51^. Plover chicks are precocial and nidifugous, and although in most species post-hatch care is provided by both parents, in others, either parent (usually females) may desert their mate during brood care to become polygamous^50,51^. Furthermore, plovers show low extra-pair paternity rates (less than 5%), indicating that social mates are a good proxy for genetic mates and thus the reproductive success of social pairs accurately reflects Darwinian fitness^52^.

In a recent study, Halimubieke et al*.*^1^ reported that snowy plovers (*Charadrius nivosus*), especially females, are more likely to divorce after successful nesting, simultaneously deserting their current brood, and initiate a new breeding attempt with a different mate, whereas, pairs tend to stay together after failed breeding attempts and initiate a second nesting attempt with the same mate. Divorcing individuals reared more offspring than those that retained their mates. This difference in mating strategy between male and female snowy plovers led to female-biased breeding dispersal, as females divorced their mates more often than males and subsequently dispersed to pursue additional mating opportunities.

**Figure 1:**
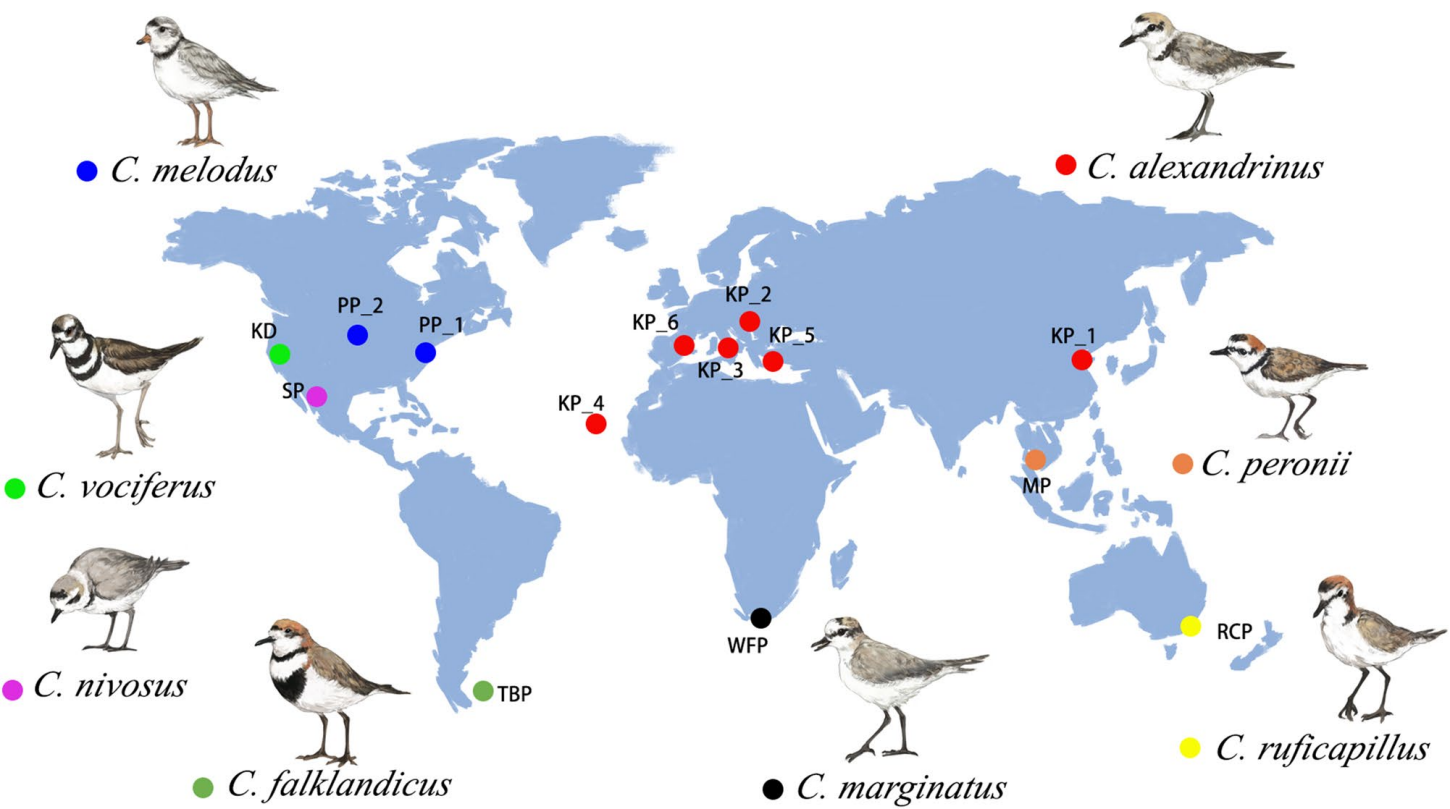
Study sites and plover populations used in this study (n = 14 populations, 8 species). Original plover illustrations and map by Siyu Ding.

Here we use data from 8 plover species across 14 populations (see Table 1 and Fig. 1 for study sites, species and study periods) to investigate four issues. First, we explore the variation in mate fidelity in both males and females across populations within breeding years, and the abiotic environmental and life history correlates of variation in mate fidelity across populations. We characterise the mean ambient temperature and temperature variation (see Methods for details), and expect that colder ambient temperature and greater temperature variation promote mate fidelity. We also investigate body weights and the sexual size dimorphism (SSD, see Methods) as life history traits, and expect that populations with heavier plovers (or less extensive SSD) show higher mate fidelity rate than populations with small plovers and more extensive SSD—based on studies that show sexual selection is associated with the extent of SSD^53,54^. Second, we evaluate the generality of the previous study in snowy plover^1^, and expect that successful breeding leads to divorce whereas failed breeding leads to mate fidelity. Third, we investigate the fitness consequences of mating decisions, and expect that birds that divorce and desert their broods have higher reproductive success than individuals that retained their mates within breeding seasons. Finally, we investigate whether mating decisions are related to breeding dispersal, as we expect that individuals that divorce their previous mate disperse greater distances to initiate another breeding attempt with a different mate than those who retain their existing mate for their next breeding attempt^1^.

**Table 1:** List of plovers *Charadrius* spp*.* populations used in the study (8 species, 14 populations).

Species	English name	Population	Code	Coordinates	Years of data collection	No. divorced male	No. divorced female	No. retention
*C. alexandrinus*	Kentish plover	Bohai Bay (China)^1^	KP_1	39º 09′ N, 118º 09′ E	2016–2018	7	5	3
*C. alexandrinus*	Kentish plover	Great Hungarian Plain (Hungary)^2,3^	KP _2	46º 40′ N, 19º 10′ E	1989–1994	4	7	16
*C. alexandrinus*	Kentish plover	Senigallia (Italy)	KP _3	43° 43′ N, 13° 12′ E	2011–2017	14	12	21
*C. alexandrinus*	Kentish plover	Maio Island (Cape Verde)	KP _4	15º 09′ N, 23º 13′ W	2009–2010; 2013–2015	3	1	13
*C. alexandrinus*	Kentish plover	Tuzla Lake (Turkey)^4^	KP _5	36º 42′ N, 35º 03′ E	1996–1999	23	26	29
*C. alexandrinus*	Kentish plover	Llobregat Delta (Spain)^5^	KP _6	41º 18′ N, 02º 08′ E	2004–2007	5	5	9
*C. melodus*	Piping plover	New Jersey (USA)	PP_1	39° 04′ N, 74° 43′ W	2012–2013; 2015–2016	3	6	48
*C. melodus*	Piping plover	Great Plain (USA)^6^	PP_2	42° 51′ N, 97° 29′ W	2005–2009; 2012–2015	17	45	44
*C. ruficapillus*	Red-capped plover	Altona (Cheetham) Saltworks (Australia)^7^	RCP	37º 53′ S, 144º 47′ E	2010–2013	1	0	16
*C. nivosus*	Snowy plover	Ceuta Bay (Mexico)^8^	SP	23º 54′ N, 106º 57′ W	2006–2012	26	79	29
*C. falklandicus*	Two-banded plover	Sea Lion Island (Falklands)^9^	TBP	51º 41′ S, 59º 10′ W	2006–2008	2	6	17
*C. vociferus*	Killdeer	Honey Lake, California (USA)^10^	KD	40° 7′ N, 120° 14′ W	1993–1997; 99–2001	18	11	106
*C. marginatus*	White-fronted plover	Cape Peninsula (South Africa)	WFP	34º 08′ S, 18º 20′ E	1999–2000; 2002–2004; 2006	0	0	439
*C. peronii*	Malaysian plover	Prachuap Khiri Khan (Thailand)^11–14^	MP	12º 00′ N, 99º 53′ E	2004–2005	1	2	9

See Appendix S2 for references for populations used in this study.

## Results

### Mate fidelity rate

**Figure 2:**
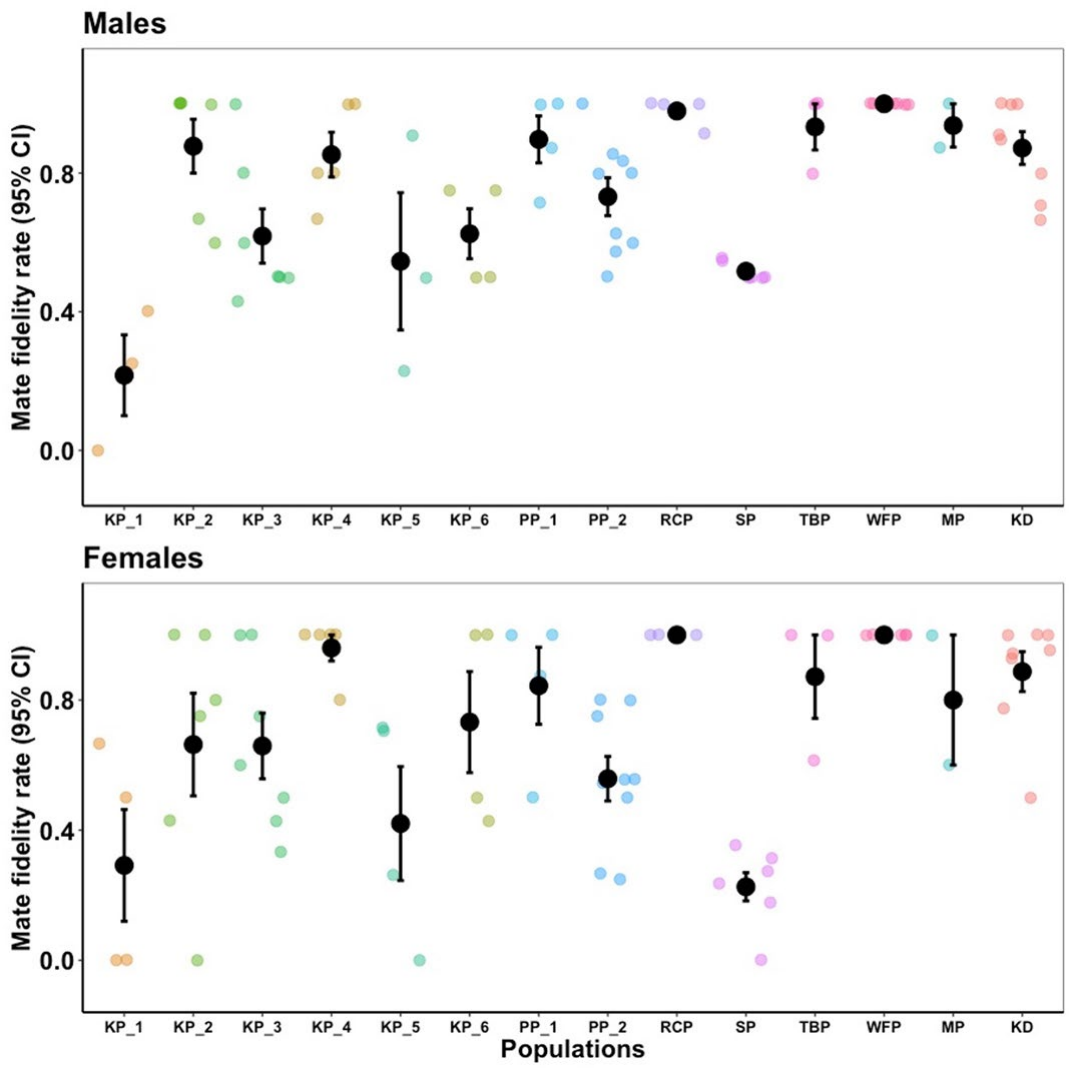
Mate fidelity rates in plover populations. Annual mate fidelity rate of each population is shown in different colours. Means of annual mate fidelity rate of each population, lower and upper 95% confidence intervals are shown. See Table 1 for details of the population codes.

Mate fidelity rates (proportion of retained individuals in given breeding season; see Methods for further details) of both males and females were different between plover populations in both sexes (see Fig. 2; male: *F* = 8.33, *df* = 13,* P* < 0.001; female: *F* = 6.34, *df* = 13, *P* < 0.001; one-way ANOVA).

### Environmental and life history predictors of mate fidelity

**Figure 3:**
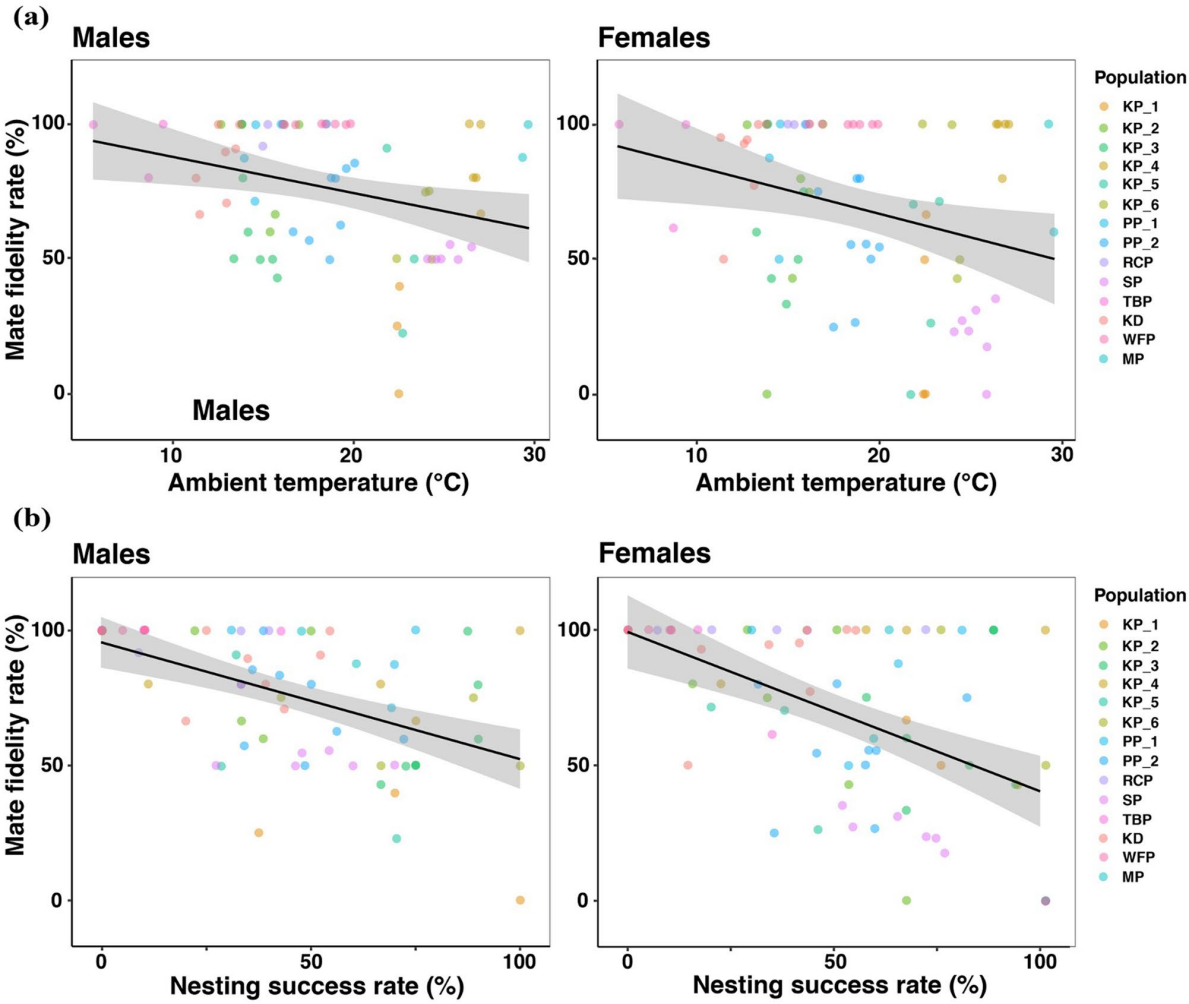
Mate fidelity rate in relation to (**a**) ambient temperature and (**b**) nesting success rate in male and female plovers (see Table 2). Annual mate fidelity rate of each population is shown in different colours. Linear regression lines are shown in black with lower and upper 95% confidence intervals (grey).

Mate fidelity rate decreased with ambient temperature (i.e., mean temperature over breeding season, see Methods; Table 2). Males (but not females) in warmer climates had lower mate fidelity than individuals that breed in colder environments (Fig. 3a). However, mate fidelity was unrelated to temperature variation (i.e., between-year fluctuations in ambient temperature, see Methods). Within populations, mate fidelity was unrelated to daily temperature in both males (*P* = 0.41, *N* = 788 observations) and females (*P* = 0.69, *N* = 776 observations; see Table S1 in the Supporting information).

**Table 2:** Mate fidelity in relation to ambient temperature, temperature variation, nesting success rate, body weight and sexual size dimorphism (SSD) in plover populations (generalised linear mixed models via Template Model Builder (TMB), including species and population as random effect variables).

Response variable	Explanatory variables	Estimate	SE	*z* value	*P* value
Mate fidelity rate in males (n = 70 years)	Intercept	8.26	2.50	3.32	** < 0.001**
	Ambient temperature	− 0.11	0.52	− 2.10	**0.04**
	Temperature variation	− 2.46	1.37	− 1.80	0.07
	Nesting success rate	− 2.53	0.70	− 3.59	** < 0.001**
	Body weight	− 0.05	0.03	− 1.52	0.13
	SSD	− 64.34	36.63	− 1.76	0.08
Mate fidelity rate in females (n = 73 years)	Intercept	5.87	4.01	1.46	0.14
	Ambient temperature	− 0.05	0.08	− 0.68	0.49
	Temperature variation	− 1.27	1.87	− 0.68	0.49
	Nesting success rate	− 2.21	0.81	− 2.72	**0.01**
	Body weight	− 0.04	0.06	0.71	0.48
	SSD	− 60.74	40.59	− 1.50	0.13

Separate models were constructed for males and females. SE = standard error. *P* values < 0.05 are emboldened.

Mate fidelity rates were unrelated to body weight or SSD between populations (Table 2; see Methods), nor within populations (male:* P* = 0.34, *N* = 136 observations; female: *P* = 0.12, *N* = 193 observations; GLMM; Table S1).

### Mate fidelity in relation to nesting success and egg-laying date

**Figure 4:**
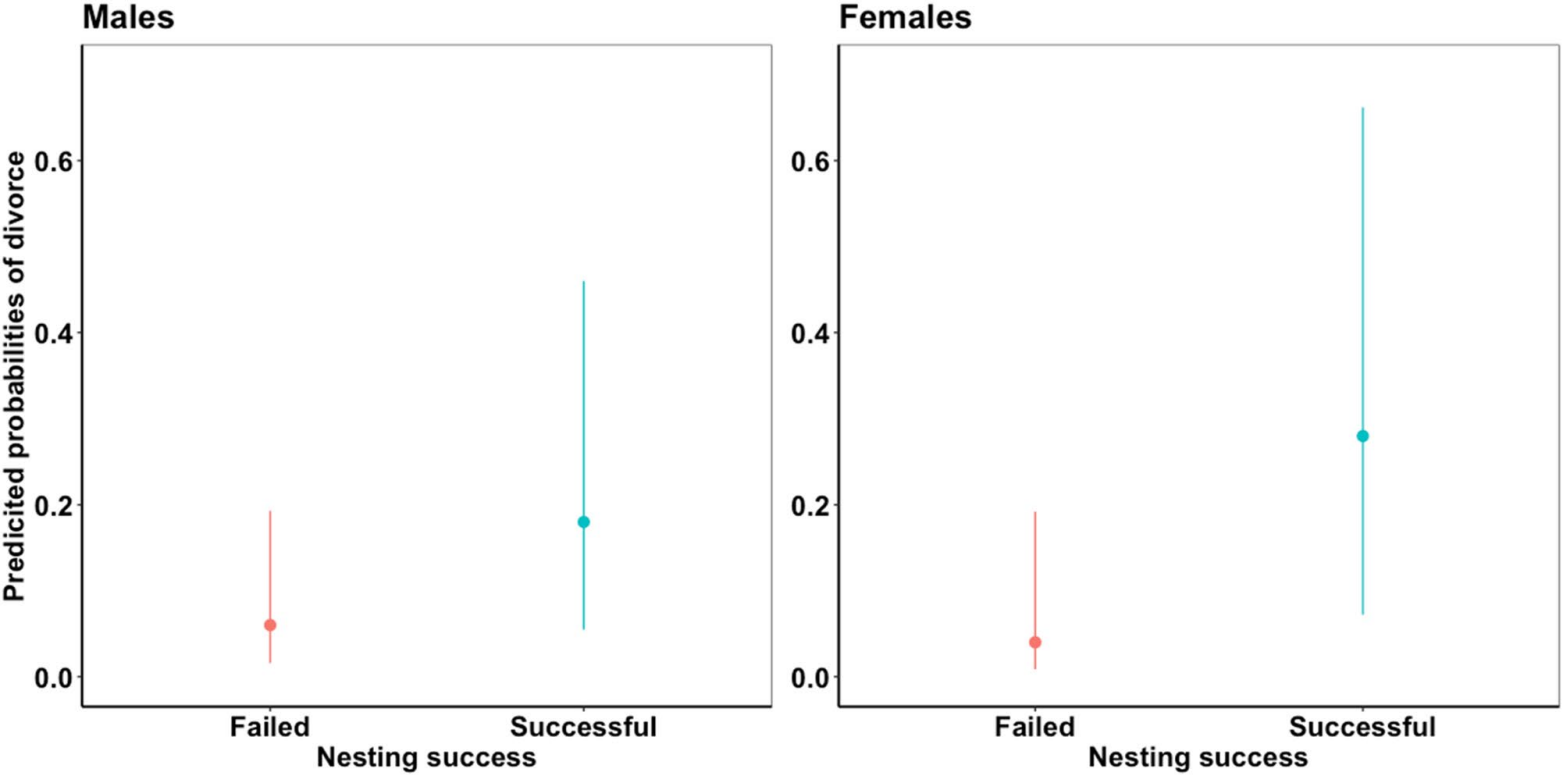
Mate fidelity in relation to nesting success in male and female plovers (see Table 3). Predicted probabilities of divorce, lower and upper 95% confidence intervals are shown.

Mate fidelity rate declined with nesting success rate (i.e., proportion of successfully hatched nests) since populations with high nesting success rates showed lower mate fidelity rate (Table 2, Fig. 3b). Consistently, within populations mate fidelity was related to nesting success, as divorce was more likely when the nest hatched successfully, whereas mate retention was more likely if the nest failed (Fig. 4; Table 3). However, egg-laying date was not significantly related to mate fidelity (Table 3).

**Table 3:** Mate fidelity in relation to nesting success and egg-laying date in plover populations.

Response variable	Explanatory variables	Estimate	SE	*z* value	*P* value
Mate fidelity in males (n = 839 observations)	Nesting success (successful)	− 1.56	0.67	− 2.32	**0.02**
	Nesting success (failed)	− 2.78	0.67	− 4.14	** < 0.001**
	Egg-laying date	− 0.18	0.21	− 0.85	0.40
Mate fidelity in females (n = 921 observations)	Nesting success (successful)	− 1.02	0.81	− 1.26	0.21
	Nesting success (failed)	− 3.14	0.83	− 3.80	** < 0.001**
	Egg-laying date	− 0.15	0.22	− 0.68	0.49

Generalized Linear mixed model with binomial family and including male/female ID, year, population and species as random effect variables. SE = standard error. *P* values < 0.05 are emboldened.

### Implications of mate fidelity

**Figure 5:**
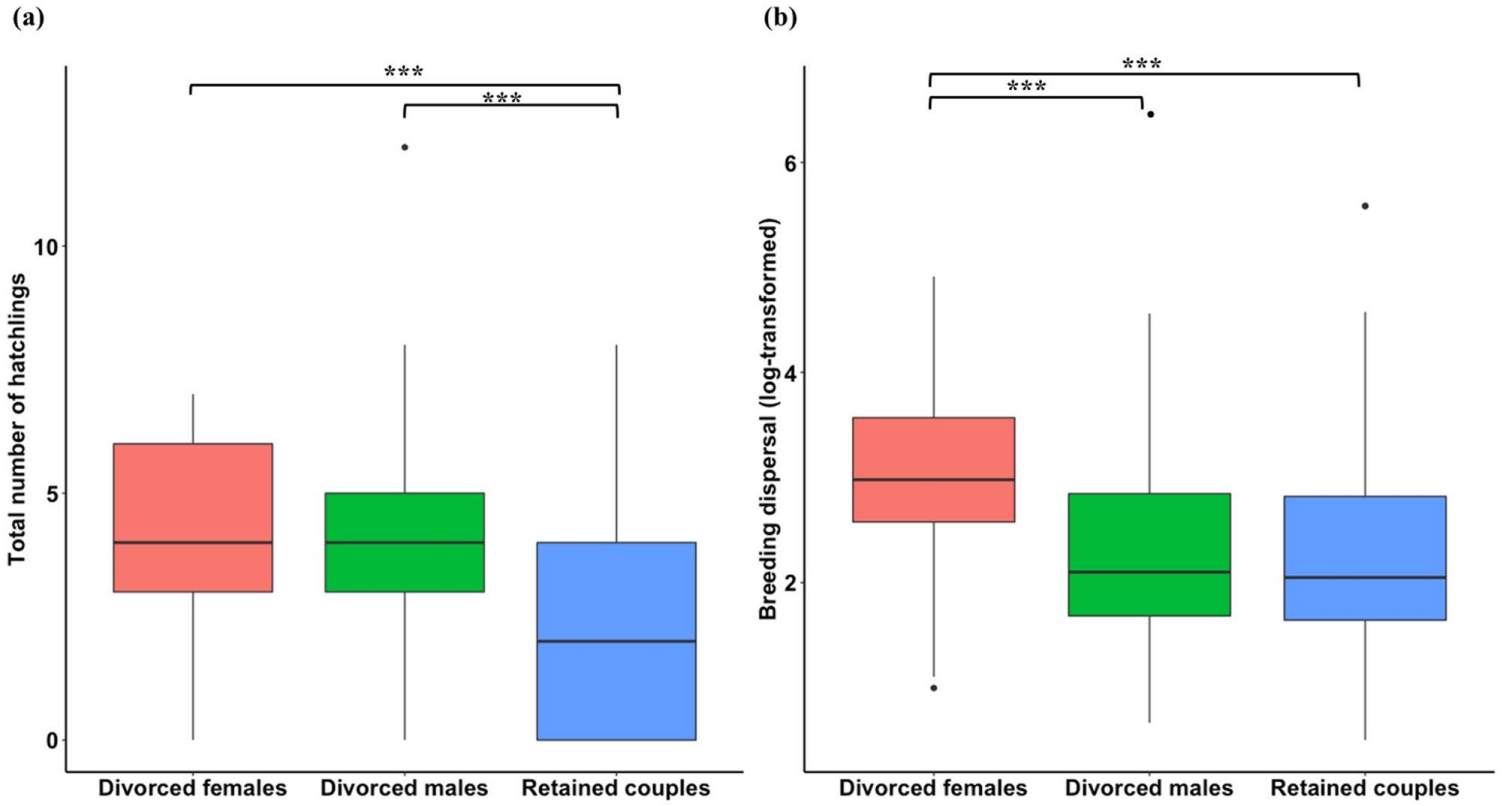
(**a**) Reproductive success and (**b**) breeding dispersal in relation to mate fidelity in plovers (see Tables 4 and 5). Breeding dispersal was estimated in meters and log-transformed (ln). Medians, upper and lower quartiles, as well as extreme values are shown, *** represents *P* < 0.001.

Divorced plovers (both males and females) produced significantly more hatchlings within years than those that retained their mate, although reproductive success (defined as the total number of hatchlings produced within a breeding year) was not different between divorced males and divorced females (Fig. 5a; Table 4). Divorced females dispersed greater distances than divorced males (breeding dispersal was defined as the straight‐line distance in meters between an individual's successive nests within a breeding season); however, divorced males did not disperse farther than retained pairs (Fig. 5b, Table 5).

**Table 4:** Comparison of reproductive success between divorced males, divorced females and retained pairs in plover populations (GLMM with Poisson error, maintaining individual ID, species and population as random effect variables, χ^2^ = 39.76, *df* = 2, *P* < 0.001, followed by *post-hoc* pairwise Tukey test).

Comparison	Estimate	SE	z ratio	*P* value
Divorced females vs. divorced males	0. 06	0.09	0.62	0.81
Divorced females vs. retained pairs	0.41	0.07	5.92	** < 0.001**
Divorced males vs. retained pairs	0.35	0.09	4.06	** < 0.001**

SE = standard error. *P* values < 0.05 are emboldened.

**Table 5:** Comparison of breeding dispersal between divorced males, divorced females and retained pairs in plover populations (LMM via REML was fitted and maintained individual ID, species and population as random effect variables, χ^2^ = 153.76, *df* = 2, *P* < 0.001, followed by *post-hoc* pairwise Tukey test).

Comparison	Estimate	SE	df	*t* ratio	*P* value
Divorced females vs. divorced males	0. 75	0.11	670	7.1	** < 0.001**
Divorced females vs. retained pairs	0.96	0.08	717	12.29	** < 0.001**
Divorced males vs. retained pairs	0.216	0.09	717	2.28	0.06

SE = standard error. *P* values < 0.05 are emboldened.

## Discussion

Three major insights have emerged from our global study. First, our results indicate that mate fidelity rates within both sexes differ among populations, consistent with previous studies of plovers^4,16,45^. For instance, Kentish plover *Charadrius alexandrinus* populations in Europe and China commonly display serial polygamy with mostly females divorcing their mate soon after the chicks hatched^55–57^; whereas the island population of Kentish plovers in Cape Verde is exclusively monogamous^58^. The social mating system of all other plover species included in our study is monogamy except for the snowy plover which exhibit serial polygamy^59,60^. Variation in mate fidelity between closely related species and populations is also common in primates, ungulates and fishes^61–63^.

Our study revealed that mate fidelity variation among plover populations is predicted by the ambient temperature, since populations in colder climates had higher mate fidelity rates than populations in warmer climates. We suggest that ambient temperature may largely influence the mate fidelity rate by its association with an increase in the time available for breeding, and by increasing the chance that at least one breeding attempt will be successful^14–16^. For example, cold environments with short breeding seasons may limit the opportunity of multiple breeding with a new mate given that mate-search and courtship are time consuming, therefore, the best strategy is to re-mate immediately with same mate if there is a breeding failure^17,32,33,64^. In contrast, mild environments with a prolonged breeding season enable a single parent to rear the offspring, and thus provide an opportunity for multiple breeding attempts for the other parent^1,19,20^. However, it is also possible that temperature may influence mate fidelity rate by directly influencing other related behaviours or physiological processes^14^. Ambient temperature appears to exert a weaker influence on mate fidelity of females than on males across populations, as males of populations from colder environments exhibit significantly higher mate fidelity rates than those from warmer environments. Further research is needed to clarify whether the different responses of males and females to environmental conditions are directly influenced by abiotic factors^65–67^, or indirectly influenced by social environment (e.g. ASR)^60^.

Our results also showed that mate fidelity rate exhibits no relationship with temperature variation between years, although studies suggest that annual fluctuations in temperature affect mating decisions in insect, reptile and mammalian species^68–70^. We argue that temperature variation is probably a crude proxy of ambient environment fluctuation, since fluctuations in other abiotic environmental factors, for example precipitation and habitat quality, may also influence mate fidelity^14,21–23^.

While we are unable to measure variation in the social environment across populations in this study, we also propose that aspects of the social environment, such as ASR, may also be strong contributor to mate fidelity variation. Recent studies show that ASR may deviate from 1:1 in a variety of organisms^26,71–73^. Variation in the ASR can alter the mating opportunities of breeding males and females, thus, influence divorcing and re-mating strategies^25,60,72^. The role of ASR influencing mating system variation in plovers and beyond will need to be revisited in the near future, although provisional studies of 4 populations suggest ASR does relate to parental care^19,60,71^.

The second major insight of our study is that breeding success is an important predictor of divorce. At the population level, populations with high nesting success rates have lower mate fidelity rates compared to populations with low nesting success rates. Consistently, individuals were more likely to divorce after clutches hatched rather than when they did not hatch, and failed breeders typically re-nested with the same partner in each population with the possible exception of white-fronted plovers, *Charadrius marginatus* in which divorce was not observed. As a consequence, divorced individuals, counterintuitively, rear more offspring compared to faithful individuals (Fig. 5a). This finding does not support the “*incompatibility hypothesis*”^37,38^, which predicts that breeding pairs with low breeding success should be more likely to divorce^32,74^. We posit that by divorcing and rapidly changing partners, while simultaneously deserting their current brood, individuals can produce more offspring within a limited season to maximize their reproductive success. Why would divorce be beneficial? (i) Offspring mortality is generally high and stochastic in shorebirds, thus individuals may need to reproduce several times within a breeding season to produce at least some offspring^75^. (ii) Chicks are precocial and only require modest care^19^, which provides the opportunity for one parent to terminate care and initiate a new clutch with another mate^76–78^. In contrast, mate retention was more likely after breeding failure. We suggest re-mating with the previous partner is the fastest way for both pair members to breed again (“*fast-track hypothesis*”^79^; reviewed by Fowler^80^; also see^32,33^); Breeding failure is related to partner compatibility in insects and mammalian species^81,82^, although we suspect that nest failure in shorebirds is majorly driven by predation^83–85^, thus we presume the role of partner compatibility in divorce is weaker compared to extrinsic forces like predation. Therefore, re-mating seems more important than changing partners and risking not finding a new mate.

Third, we found that mate fidelity is related to breeding dispersal. After divorce, female plovers disperse significantly farther than males. Sex‐biased dispersal has been well-documented in invertebrates, reptiles, birds, and mammals, and it is proposed to be related to mating system^41–43^. For example, many mammals are socially polygynous, males do not participate in parental care, females rely on home ranges with resources to successfully rear offspring, therefore, male-biased dispersal is expected. Whereas in birds, which are typically socially monogamous, males demonstrate territorial defence behaviour because high quality breeding sites provide good resources and opportunities for successful breeding, thus reducing male’s tendency to move large distance between breeding attempts^86–89^. Our result follows the general pattern of female-biased breeding dispersal observed in most bird species including shorebirds^90,91^. Additionally, we propose that for polygamous populations there is an additional reason: females have the opportunity to desert the brood and seek a new mate from within a wide geographical area^44^.

Taken together, our results illustrate that (i) mating decisions are associated with the abiotic environmental conditions; (ii) birds that divorce and desert their broods generally attain higher breeding success than individuals that retained their mates; and (iii) the asymmetric mating opportunities of males and females result in different spatial dispersal patterns. Our results support the proposition that divorce is a strategy employed to improve reproductive success^1,55,92^. We suggest that divorce is an adaptive response to environmental constraints (e.g. limited breeding time), life history traits (e.g. low survival rate of the young, uniparental care) and population demography (e.g. biased ASR). We call for further studies to build upon our research framework by augmenting these analyses with other more environmental variables (e.g. precipitation) and incorporating information on the social environment (e.g. ASR) and broader scope of life history traits (e.g. survival rate, longevity). In addition, we encourage the development of theoretical models investigating the influence of ecological/ social environment and life history on the evolution of breeding systems.

## Methods

### Study site and fieldwork

Fieldwork was carried out in 14 breeding populations of 8 plover species and ranged from 1 to 9 breeding seasons per population (see Table1 and Fig. 1 for study sites and study periods). Egg-laying date of nests was either known (for nests that were found during egg-laying) or estimated by floating eggs or measuring egg mass relative to egg size^26,93^. Breeding pairs were captured on their nest while incubating eggs, using funnel traps, noose mats, box traps or bownet traps. Morphological data of each individual were collected: body weight was collected from eight populations (body weight data from six populations were not collected in the field, see below for details); sex was determined by morphological features, for monomorphic species, molecular sexing was applied to identify the sex of the individual. Finally, each individual was banded with a unique combination of colour rings/flags and a metal ring (see Appendix S2 in Supplementary Material and further references in^93^). Nests were monitored until hatching to obtain nesting success data (see below for details).

### Data collection

#### Quantification of mate fidelity and mate fidelity rate within breeding years

Plovers that were included in this study could freely retain or divorce their mates in natural conditions without manipulation. The mating decision of each individual was recorded as either *mate retention* or *divorce* with respect to their previous breeding attempt within each breeding year. The mating decision of males and females were not independent from each other, therefore, we assessed mating decisions separately for banded males and females in each population. We used the same criteria, following Halimubieke et al*.*^1^, for individuals that were included in the analyses: (a) the identities of the individuals and their mate(s) were known, (b) they were observed in at least two reproductive attempts within a breeding year, and (c) if there was a mate change, only those who changed their mates while the previous mate was known to be alive were included. In total, 1927 breeding events (Table 1, 124 divorces in males, 205 divorces in females, and 799 retentions in both sex) fitted the criteria for the mate fidelity analysis in the 14 populations. Mate fidelity rate represents the proportion of retained individuals in given breeding year(s) in each population.

#### Abiotic environment, body weight and sexual size dimorphism (SSD)

In this study, the ambient temperature and temperature variation of each population over the study period are used as the proxies of abiotic environmental conditions. We extracted high resolution historical daily temperature data collected by the nearest weather stations for each study site from the National Oceanic and Atmospheric Administration (NOAA) database and University of East Anglia Climate Research Unit database (CRU; https://www.cru.uea.ac.uk/, version 3.10.01), using the R package “rnoaa”^94^. The average distance between weather stations and study sites is 60.17 km (see Table S2 for more details). If the weather record was incomplete for any study site, we used the R package “GSODR”^95^ to extract weather data from the USA National Center for Environmental Information (NCEI) database. Since our study focused on breeding behaviour, we only extracted daily temperatures from each month of the breeding season(s) when capture data were collected in a given population. and we calculated the average temperature of the breeding season(s); we refer to this variable as ambient temperature. The temperature variation refers to average between-year fluctuations in ambient temperature, and it was calculated as the standard deviation of the average temperature of all breeding years for a given population.

The average body weight of birds from each population was calculated from individual body weight data collected from the fieldwork over the study period. We also searched for average body weight data in Handbook of the Bird of the World^51^ and CRC Handbook of Avian Body Masses^96^ for the following populations for which we did not have field data: the Kentish plover population from Italy, two piping plover populations from USA, the red-capped plover population, the white-fronted plover population and the Malaysian plover population*.* To quantify sexual size dimorphism, we divided the male average body weight by that of the female and log-transformed this ratio, and assigned positive signs when males were the larger sex and negative ones when females were larger.

#### Nesting success and egg-laying date

Nesting success was determined based on the fate of the nest(s) of each individual included in our study. The fate of a nest was recorded as either successful (at least one chick hatched) or failed (no chicks hatched due to predation, destruction, abandonment, eggs disappeared < 15 days after estimated laying date, eggs did not hatch, or the nest was flooded). The nesting success rate represents the proportion of nests with at least one successfully hatched egg in each population over the study period. The egg-laying date was used to quantify breeding phenology. We controlled for breeding phenological differences between years by converting egg‐laying dates into Julian dates (“lubridate” package in R^97^), and standardised egg-laying date using the z‐transformation (mean = 0, SD = 1).

#### Reproductive success and breeding dispersal

Reproductive success was quantified as the cumulative number of hatchlings each individual produced in all breeding attempts within each breeding year. We did not use the number of fledglings as the proxy for reproductive success as the fates of fledglings are difficult to estimate in precocial species like plovers due to the high mobility and camouflage of broods^16^. Breeding dispersal was defined as the straight‐line distance (in meters) between an individual's successive nests within a year for those populations with nest location data.

### Statistical analyses

To investigate variation in mate fidelity rate across populations, first, we used analysis of variance ANOVA to compare the mate fidelity rates of both sexes across 14 populations. We then constructed two generalised linear mixed models (GLMM) via Template Model Builder (TMB) with binomial error structure to test environmental and life history predictors of mate fidelity rate in both sexes. In these models, the mate fidelity rate (male or female) of each population over the study period was the dependent variable. Ambient temperature, temperature variation and nesting success rate of each population over the study period, alongside average body weight (male or female) and SSD of the populations were used as explanatory variables. Species and population were included as random effects.

To investigate individual mating decisions, we first constructed a GLMM for each sex with a binomial error structure, and examined how mate fidelity relates to nesting success and relative egg-laying date. A similar model for each sex was developed to explore mate fidelity in relation to daily temperature and individual body size. In these models, species, population, individual identity and year were used as random effect variables.

Next, we used a GLMM to investigate if reproductive success is related to mate fidelity by comparing the total number of hatchlings from all clutches among divorced males, divorced females and retained pairs. A Poisson error structure was used because: (i) Gaussian version of the model suggested normality assumptions were violated; (ii) reproductive success is a count and thus an integer variable. Species, population and individual identity were used as random effects.

To investigate the relationship between breeding dispersal and mate fidelity, we use same methods as Halimubieke et al*.*^1^. We built a linear mixed-effects model (LMM) using log-transformed (ln) breeding dispersal as the dependent variable, and mate fidelity groups (divorced males, divorced females and retained pairs) as the explanatory variable. LMM via REML was fitted and included population and individual identity as random effect variables. The goodness-of-fit test showed that the residuals of the model show equal variances and follow normal distribution, supporting the validation of the model we used.

Estimated marginal means (emmeans from package “emmeans” in R^98^) were calculated for each group in the latter two models, and *post-hoc* pairwise comparisons adjusted by Tukey were applied to test group differences. There was no model simplification and all terms were retained in all the models above.

To test whether phylogenetic relatedness influenced our results, we followed the same method as Vincze et al*.*^99^, the above models were repeated using Bayesian Markov chain Monte Carlo GLMM (MCMCglmm^100^), including a correlational structure based on the species-level phylogenetic tree of the 8 *Charadrius* species studied here. The phylogenetic signal of the investigated trait in these models was low (model description and calculation of the phylogenetic signal are given in Appendix S1). All statistical analyses were performed using R version 3.5.1^101^.

### Ethical statement

This study did not involve any manipulation experiments, and all methods were carried out in accordance with relevant guidelines and regulations of each country in which it was performed. Fieldwork and bird-ringing procedures were authorized by relevant authorities: Hungary (Environmental Ministry and Kiskunság National Park); Australia (Deakin University Animal Welfare Committee Permits B02-2012, B20-2014 and B10-2016, State Government Permits 10006205, 10007918 and 10007241 and Australian Bird and Bat Banding Scheme (ABBBS) Authorities 1763, 3271 and 3033); Mexico (#SGPA/ DGVS/01717/10, #SGPA/DGVS/01367/11), Spain (Ministry of Environment #660117); California (U.S. Fish and Wildlife (USFWS) #TE807078 and U. S. Geological Survey (USGS) #09316); Great Plain, USA (the U.S. Geological Survey Bird Banding Laboratory with Federal Master Bander permit #21446 with threatened and endangered species endorsements, Federal Threatened and Endangered Species handling permit#TE103272-3, and IACUC protocol #14-003.); China (Hebei Forestry Bureau); South Africa (Cape Nature and SAFRING); Cape Verde (Directorate Geral Ambiente); Turkey (Turkish Ministry of National Parks, Tuzla Municipality and Governor of Karatas, Mr. E. Karakaya).

## Supplementary information

Supplementary Information 1.

## Data Availability

All data will be archived at Dryad once the manuscript is accepted for publication. Relevant data is available from GitHub (https://github.com/narhulan29/charadriusplovers).
