# Author Correction: Successful breeding predicts divorce in plovers

**DOI:** 10.1038/s41598-021-81033-w

**Published:** 2021-01-07

**Authors:** Naerhulan Halimubieke, Krisztina Kupán, José O. Valdebenito, Vojtěch Kubelka, María Cristina Carmona-Isunza, Daniel Burgas, Daniel Catlin, James J. H. St Clair, Jonathan Cohen, Jordi Figuerola, Maï Yasué, Matthew Johnson, Mauro Mencarelli, Medardo Cruz-López, Michelle Stantial, Michael A. Weston, Penn Lloyd, Pinjia Que, Tomás Montalvo, Udita Bansal, Grant C. McDonald, Yang Liu, András Kosztolányi, Tamás Székely

**Affiliations:** 1 Milner Centre for Evolution, Department of Biology and Biochemistry, University of Bath, Bath, UK; 2 Behaviour Genetics and Evolutionary Ecology Research Group, Max Planck Institute for Ornithology, Seewiesen, Germany; 3 Department of Evolutionary Zoology and Human Biology, University of Debrecen, Debrecen, Hungary; 4 Department of Animal and Plant Sciences, University of Sheffield, Alfred Denny Building, Western Bank, Sheffield, UK; 5 Department of Biodiversity Research, Global Change Research Institute, Czech Academy of Sciences, Brno, Czech Republic; 6 Departamento de Ecología Evolutiva, Instituto de Ecología, Universidad Nacional Autónoma de México, Ciudad de México, México; 7 Department of Biological and Environmental Science, University of Jyväskylä, Jyväskylä, Finland; 8 Department of Fish and Wildlife Conservation, Virginia Tech, Blackburg, USA; 9 Centre for Biological Diversity, School of Biology, University of St Andrews, St Andrews, UK; 10 Department of Environmental and Forest Biology, SUNY College of Environmental Science and Forestry, Syracuse, USA; 11 Department of Wetland Ecology, Estación Biológica de Doñana, Sevilla, Spain; 12 Quest University Canada, Squamish, Canada; 13 Forest Supervisor’s Office, USDA Forest Service, Plumas National Forest, Quincy, CA USA; 14 Associazione ARCA, Senigallia‑Anoca, Italy; 15 Posgrado en Ciencias del Mar Y Limnología, Universidad Nacional Autónoma de México, Ciudad Universitaria, Cd. México Mexico; 16 School of Life and Environmental Sciences, Faculty of Science, Engineering and the Built Environment, Deakin University, Burwood, Australia; 17 FitzPatrick Institute, DST/NRF Centre of Excellence, University of Cape Town, Cape Town, South Africa; 18 Ministry of Education Key Laboratory for Biodiversity Science and Ecological Engineering, College of Life Sciences, Beijing Normal University, Beijing, China; 19 Chengdu Research Base of Giant Panda Breeding, Chengdu, China; 20 Sichuan Key Laboratory of Conservation Biology for Endangered Wildlife, Chengdu, China; 21 Sichuan Academy of Giant Panda, Chengdu, China; 22 Servei de Vigilancia I Control de Plagues Urbanes, Agencia de Salud Pública de Barcelona, Barcelona, Spain; 23 Centre for Ecological Sciences, Indian Institute of Science, Bengaluru, India; 24 Department of Ecology, University of Veterinary Medicine Budapest, Budapest, Hungary; 25 Department of Zoology, Edward Grey Institute, University of Oxford, Oxford, UK; 26 State Key Laboratory of Biocontrol, School of Ecology/School of Life Sciences, Sun Yat-Sen University, Shenzhen, China

Correction to: *Scientific Reports* 10.1038/s41598-020-72521-6, published online 23 September 2020

This Article contains errors in the Data availability section.

“All data will be archived at Dryad once the manuscript is accepted for publication. Relevant data is available from GitHub (https://github.com/narhulan29/charadriusplovers).”

should read:

“Relevant data is available in the following GitHub repository: https://github.com/Charadriusplover/Halimubieke.git.”

