# Supplementary material for: Successful breeding predicts divorce in plovers
Source: Sci Rep. 2020 Sep 23;10:15576. doi: 10.1038/s41598-020-72521-6 (PMC7511398; doi:10.1038/s41598-020-72521-6)
Supplement: Supplementary file 1 — Supplementary Information 1. [file 41598_2020_72521_MOESM1_ESM.docx]

**Successful breeding predicts divorce in plovers**

Naerhulan Halimubieke^1*^, Krisztina Kupán^2^, José O. Valdebenito^1^, Vojtěch Kubelka^1,3-5^, María Cristina Carmona-Isunza^1,6^, Daniel Burgas^7^, Daniel Catlin^8^, James J. H. St Clair^9^, Jonathan Cohen^10^, Jordi Figuerola^11^, Maï Yasué^12^, Matthew Johnson^13^, Mauro Mencarelli^14^, Medardo Cruz-López^15^, Michelle Stantial^10^, Michael A. Weston^16^, Penn Lloyd^17^, Pinjia Que^18-21^, Tomás Montalvo^22^, Udita Bansal^23^, Grant C. McDonald^24,25^, Yang Liu^26^, András Kosztolányi^24^, Tamás Székely^1,3,18,26^

^1^ Milner Centre for Evolution, Department of Biology and Biochemistry, University of Bath, Bath, UK

^2^Max Planck Institute for Ornithology, Behaviour Genetics and Evolutionary Ecology Research Group, Seewiesen, Germany

^3^Department of Evolutionary Zoology and Human Biology, University of Debrecen, Debrecen, Hungary

^4^ Department of Animal and Plant Sciences, University of Sheffield, Alfred Denny Building, Western Bank, Sheffield, UK

^5^ Department of Biodiversity Research, Global Change Research Institute, Czech Academy of Sciences, Brno, Czech Republic

^6^ Departamento de Ecología Evolutiva, Instituto de Ecología, Universidad Nacional Autónoma de México, Ciudad de México, México

^7^ Department of Biological and Environmental Science, University of Jyväskylä, Jyväskylä, Finland

^8^ Department of Fish and Wildlife Conservation, Virginia Tech, Blackburg, USA

^9^ Centre for Biological Diversity, School of Biology, University of St Andrews, St Andrews, UK

^10^ Department of Environmental and Forest Biology, SUNY College of Environmental Science and Forestry, Syracuse, USA

^11^ Department of Wetland Ecology, Estación Biológica de Doñana, Sevilla, Spain

^12^ Quest University Canada, Squamish, Canada

^13^ USDA Forest Service, Plumas National Forest, Forest Supervisor's Office, Quincy CA, USA

^14^ Associazione ARCA, Senigallia-Anoca, Italy

^15^ Posgrado en Ciencias del Mar y Limnología, Universidad Nacional Autónoma de México, Ciudad Universitaria, Cd. México, Mexico

^16^ School of Life and Environmental Sciences, Faculty of Science, Engineering and the Built Environment, Deakin University, Burwood, Australia

^17^ FitzPatrick Institute, DST/NRF Centre of Excellence, University of Cape Town, South Africa

^18^ Ministry of Education Key Laboratory for Biodiversity Science and Ecological Engineering, College of Life Sciences, Beijing Normal University, Beijing, China

^19^ Chengdu Research Base of Giant Panda Breeding, Chengdu, China

^20^ Sichuan Key Laboratory of Conservation Biology for Endangered Wildlife, Chengdu, China

^21^ Sichuan Academy of Giant Panda, Chengdu, China

^22^ Servei de Vigilancia i Control de Plagues Urbanes, Agencia de Salud Pública de Barcelona, Barcelona, Spain

^23^ Centre for Ecological Sciences, Indian Institute of Science, Bengaluru, India

^24^ Department of Ecology, University of Veterinary Medicine Budapest, Budapest, Hungary

^25^ Edward Grey Institute, Department of Zoology, University of Oxford, Oxford, UK

^26^ State Key Laboratory of Biocontrol, School of Ecology/School of Life Sciences, Sun Yat-sen University, Shenzhen, China

* Corresponding authors: Naerhulan Halimubieke, [hn364@bath.ac.uk](mailto:hn364@bath.ac.uk)

**SUPPLEMENTARY MATERIALS**

**Supplementary Acknowledgements**

VK was supported by the Ministry of Education, Youth and Sports of the Czech Republic, grant No. CZ.02.2.69/0.0/0.0/19_074/0014459. PQ was supported by the National Natural Science Foundation of China, grant No. 31600297 and 31572288. YL was supported by the Open Fund of Key Laboratory of Biodiversity Science and Ecological Engineering, Ministry of Education, China. JOV was supported by Comisión Nacional de Investigación Científica y Tecnológica (CONICYT), BECAS CHILE 72170569. MCC-I was supported by DGAPA-UNAM postdoctoral scholarship. DC was supported by U.S. Army Corps of Engineers. MY was supported by PGS-D NSERC Canada and P. Dearden’s SSHRC, Canada research grant. MAW was supported by ANZ Holsworth Research Endowment and BirdLife Australia’s beach-nesting birds project funding. MAW thanks Daniel Lees, Beach Ecology and Research Hub and a SEBE International Collaboration Grant. MJ’s funding came via grants to S. Haig and L. Oring from the U.S. Geological Survey and to L. Oring via Dan Brimm and the Nevada Agricultural Experiment Station, University of Nevada. MM was supported by ARCA association.

**Appendix S1.** Description of the Bayesian modelling framework.

To test how phylogenetic relatedness might influence our results we constructed Bayesian MCMCglmm models implemented in R package 'MCMCglmm' with categorical trait distribution^1^.The phylogenetic tree was obtained from dos Remedios et al.^2^. Since prior information about parameter distribution was not available, we used non-informative priors. All models were run for 500,000 times with a burn-in of 1,000 and a thinning interval of 500. Phylogenetic effect was tested by removing the phylogeny regarding the species random term, while keeping the latter random term in the model. Species effect was tested by removing phylogeny information and the species random term. Phylogenetic signal was calculated for all four initial models.

Prior specifications are given below, where *n* stands for the number of fixed parameters estimated in each of the models, *Species* is a phylogenetic tree representing the evolutionary history of the 8 *Charadrius* species included in this study. Number of *G* structure elements in the prior was modified according to the number of random factors in each model.

*Prior<- list (G = list (G1 = list (V = 1, nu = 1000, alpha.mu = 0, alpha.V = 1),*

*G2 = list (V = 1, nu = 1000, alpha.mu = 0, alpha.V = 1),*

*… Gn = list (V = 1, nu = 1000, alpha.mu = 0, alpha.V = 1),*

*R = list (V = n, fix = TRUE))*

*inv.phylo <-inverseA (Species,nodes = "TIPS",scale = TRUE)*

*phylogenetic signal <- model$VCV[, "species"]/(model$VCV[, "species"] + model$VCV[, "units"])*

*model 1<-MCMCglmm (mate fidelity rate ~ ambient temperature + temperature variation + nesting success rate + average body weight + SSD,*

*random = ~ species + population,*

*family = "gaussian",*

*ginverse = list (Species = inv.phylo$Ainv),*

*nitt = 500000, burnin = 1,000, thin = 500,*

*prior = Prior, singular.ok = T)*

*phylogenetic signal: 0.04*

*model 2 <-MCMCglmm (mate fidelity rate ~ ambient temperature + temperature variation + nesting success rate + average body weight + SSD,*

*random = ~ species + population,*

*family = "gaussian",*

*ginverse = list (Species = inv.phylo$Ainv),*

*nitt = 500000, burnin = 1,000, thin =500,*

*prior = Prior, singular.ok = T)*

*phylogenetic signal:* *0.04*

*model 3 <-MCMCglmm (mate fidelity ~ nesting success + egg-laying date,*

*random = ~ year + male_ID + species + population,*

*family = "categorical",*

*ginverse = list (Species = inv.phylo$Ainv),*

*nitt = 1,000,000, burnin = 1,000, thin = 500,*

*prior = Prior, singular.ok = T)*

*phylogenetic signal:* *0.38*

*model4 <-MCMCglmm (mate fidelity ~ nesting_success + egg_laying_date,*

*random = ~ year + female_ID + species + population,*

*family = "categorical",*

*ginverse = list (Species = inv.phylo$Ainv),*

*nitt = 1,000,000, burnin = 1,000, thin = 500,*

*prior = Prior, singular.ok = T)*

*phylogenetic signal:* *0.36*

*model 5<-MCMCglmm(mate fidelity ~ daily temperature*

*random = ~ year + male_ID + species + population,*

*family = "categorical",*

*ginverse = list (Species = inv.phylo$Ainv),*

*nitt= 1,000,000, burnin = 1,000, thin= 500,*

*prior = Prior, singular.ok = T)*

*phylogenetic signal:* *0.67*

*model 6<-MCMCglmm(mate fidelity ~ daily temperature*

*random = ~ year + female_ID + species + population,*

*family = "categorical",*

*ginverse = list (Species = inv.phylo$Ainv),*

*nitt = 1,000,000, burnin = 1,000, thin = 500,*

*prior = Prior, singular.ok = T)*

*phylogenetic signal:* *0.61*

*model 7<-MCMCglmm(mate fidelity ~ body weight*

*random = ~ year + male_ID + species + population,*

*family = "categorical",*

*ginverse = list (Species = inv.phylo$Ainv),*

*nitt = 1,000,000, burnin = 1,000, thin = 500,*

*prior = Prior, singular.ok = T)*

*phylogenetic signal:* *0.28*

*model 8<-MCMCglmm(mate fidelity ~ body weight*

*random = ~ year + female_ID + species + population,*

*family = "categorical",*

*ginverse = list (Species = inv.phylo$Ainv),*

*nitt = 1,000,000, burnin = 1,000, thin = 500,*

*prior = Prior, singular.ok = T)*

*phylogenetic signal:* *0.35*

**References**

1. Hadfield, J. D. MCMC methods for multi-response generalized linear mixed models: The MCMCglmm R package. *J. Stat. Softw.* **33,** 1-22; 10.18637/jss.v033.i02 (2010).

2. dos Remedios, N., Lee, P. L. M., Burke, T., Székely, T. & Küpper, C. North or south? Phylogenetic and biogeographic origins of a globally distributed avian clade. *Mol. Phylogenet. Evol.* **89,** 151-159; 10.1016/j.ympev.2015.04.010 (2015).

**Appendix S2.** Supplementary references for Table 1

1. Que, P. Breeding ecology of Kentish plover (*Charadrius alexandrinus*) at Bohai Bay, China. (Unpublished reports, 2016-2019).

2. Székely, T. & Lessells, C. M. Mate change by Kentish plovers *Charadrius alexandrinus*. *Ornis. Scand.* **24,** 317-322 (1993).

3. Székely, T. & Williams, T. D. Factors affecting timing of brood desertion by female Kentish plovers *Charadrius alexandrinus*. *Behaviour* **130,**17-28 (1994).

4. Kosztolányi, A., Székely, T., Cuthill, I. C., Yilmaz, K. T. & Berberoǧlu, S. Ecological constraints on breeding system evolution: the influence of habitat on brood desertion in Plover. *J. Anim. Ecol.* **75,** 257-265 (2006)

5. Figuerola, J. & Cerdà, F. Evolució i conservació de la població de corriol camanegre (*Charadrius alexandrinus*) del delta del Llobregat. *Spartina* **3,** 161-169 (1998).

6. Hunt, K. L. *et al*. Using nest captures and video cameras to estimate survival and abundance of breeding Piping Plovers *Charadrius melodus*. *Ibis* **162**, 1-12. doi:10.1111/ibi.12726 (2020).

7. Lomas, S. C. *et al*. The influence of cover on nesting red−capped plovers: a trade-off between thermoregulation and predation risk? *Vic. Nat.* **131,** 115-127 (2014).

8. Halimubieke, N. *et al*. Mate fidelity in a polygamous shorebird, the snowy plover (*Charadrius nivosus*). *Ecol. Evol.* **9,** 10734-10745; doi: 10.1002/ece3.5591 (2019).

9. St Clair, J. J. H., Herrmann, P., Woods, R. W. & Székely, T. Female−biased incubation and strong diel sex−roles in the two−banded plover *Charadrius falklandicus*. *J. Ornithol.* **151,** 811-816. doi: 10.1007/s10336-010-0517-9 (2010).

10. Johnson, M., Oring, L. W. & Walters, J. R. Killdeer parental care when either parent deserts. *WSGB* **110,** 43-47 (2006).

11. Yasué, M. & Dearden, P. The effects of heat stress, predation risk and parental investment on Malaysian plover nest return times after a human disturbance. *Biol. Conserv.* **132,** 472-480. doi: 10.1016/j.biocon.2006.04.038 (2006).

12. Yasué, M. & Dearden, P. Simultaneous biparental incubation of two nests by a pair of Malaysian plovers. *WSGB* **109,** 121-122 (2006).

13. Yasué, M. & Dearden, P. Constraints on successive clutching behaviour of sedentary Malaysian plovers breeding in a tropical environment. *Ardea* **96,** 59-72 (2007).

14. Yasué, M. & Dearden, P. Sex−roles of Malaysian plovers *Charadrius peronii* during territory acquisition, incubation and brood care. *J. Ethol.* **26,** 99-112 (2007).

**Table S1.** Mate fidelity in relation to daily temperature and body weight within breeding years in plover populations. Generalised linear mixed model with binomial family and including male/female ID, year, population and species as random effect variables. SE = standard error. *P* values < 0.05 are emboldened.

| **Response variable** |  | **Explanatory variable** | **Estimate** | **SE** | ***z* value** | ***p* value** |  |
| --- | --- | --- | --- | --- | --- | --- | --- |
| **Mate fidelity in males (n = 788 observations)** | | | | | | | |
|  |  | Intercept | -2.58 | 1.12 | -2.17 | **0.03** |  |
|  |  | Daily temperature | -0.03 | 0.03 | -0.82 | 0.41 |  |
| **Mate fidelity in females (n = 776 observations)** | | | | | | | |
|  |  | Intercept | -3.86 | 1.78 | -2.16 | **0.03** |  |
|  |  | Daily temperature | 0.01 | 0.03 | 0.40 | 0.69 |  |
| **Mate fidelity in males (n = 136 observations)** | | | | | | | |
|  |  | Intercept | 1.12 | 1.67 | 0.67 | 0.50 |  |
|  |  | Body weight | -0.03 | 0.04 | -0.95 | 0.34 |  |
| **Mate fidelity in females (n = 193 observations)** | | | | | | | |
|  |  | Intercept | 2.51 | 1.79 | 1.40 | 0.16 |  |
|  |  | Body weight | -0.06 | 0.04 | -1.55 | 0.12 |  |

**Table S2. Summary description of the distances of weather stations from study sites (km) used in the study.**

| **English name** | **Population** | **Distance of weather stations from study site (km)** |
| --- | --- | --- |
| Kentish plover | Bohai Bay (China) | 29 |
| Kentish plover | Great Hungarian Plain (Hungary) | 19.2 |
| Kentish plover | Senigallia (Italy) | 202 |
| Kentish plover | Maio Island (Cape Verde) | 181 |
| Kentish plover | Tuzla Lake (Turkey) | 204 |
| Kentish plover | Llobregat Delta (Spain) | 4.5 |
| piping plover | New Jersey (USA) | 21 |
| piping plover | Great Plain (USA) | 48.1 |
| red-capped plover | Altona (Cheetham) Saltworks (Australia) | 5.1 |
| snowy plover | Ceuta Bay (Mexico) | 12 |
| two-banded plover | Sea Lion Island (Falklands) | 5 |
| killdeer | Honey Lake, California (USA) | 21.3 |
| white-fronted plover | Cape Peninsula (South Africa) | 50 |
| Malaysian plover | Prachuap Khiri Khan (Thailand) | 40.2 |
